# Allosteric Pathways in the PPARγ-RXRα nuclear receptor complex

**DOI:** 10.1038/srep19940

**Published:** 2016-01-29

**Authors:** Clarisse G. Ricci, Rodrigo L. Silveira, Ivan Rivalta, Victor S. Batista, Munir S. Skaf

**Affiliations:** 1 Institute of Chemistry, University of Campinas-UNICAMP, Cx. P. 6154, Campinas SP 13084-862, Brazil; 2 Université de Lyon, CNRS, Laboratoire de Chimie, École Normale Supérieure de Lyon, UMR 5182, 46 Allée d’Italie, 69364 Lyon, Cedex 07, France; 3 Department of Chemistry, Yale University, P.O. Box 208107, New Haven, CT 06520-8167, United States

## Abstract

Understanding the nature of allostery in DNA-nuclear receptor (NR) complexes is of fundamental importance for drug development since NRs regulate the transcription of a myriad of genes in humans and other metazoans. Here, we investigate allostery in the peroxisome proliferator-activated/retinoid X receptor heterodimer. This important NR complex is a target for antidiabetic drugs since it binds to DNA and functions as a transcription factor essential for insulin sensitization and lipid metabolism. We find evidence of interdependent motions of Ω-loops and PPARγ-DNA binding domain with contacts susceptible to conformational changes and mutations, critical for regulating transcriptional functions in response to sequence-dependent DNA dynamics. Statistical network analysis of the correlated motions, observed in molecular dynamics simulations, shows preferential allosteric pathways with convergence centers comprised of polar amino acid residues. These findings are particularly relevant for the design of allosteric modulators of ligand-dependent transcription factors.

Understanding the molecular origin of allosteric mechanisms in protein and DNA complexes is a challenge of great current interest^1^,^2^ because of its importance to cellular regulation and the design and development of allosteric drugs^3^. Despite recent efforts^4^,^5^,^6^,^7^,^8^, allostery remains a biophysical enigma since little is known about the molecular mechanisms that trigger long-range conformational changes in biomolecular systems^9^,^10^. However, it is well known that fluctuations between conformational substates^11^,^12^ are key for activation and functional regulation under various physiological conditions^13^. Here, we focus on the molecular origin of allosteric pathways in nuclear receptors (NRs) that regulate the transcription of a myriad of genes in humans and other metazoans^14^,^15^.

NRs are proteins that attach to specific sequences of DNA known as Hormone Response Elements (HRE), forming homodimers or heterodimers with the promiscuous Retinoic X Receptor (RXR), depending on the NR family type^14^,^16^. Each NR monomer comprises three major domains: (i) the amino terminus domain (also known as the A/B region) containing a transactivation domain; (ii) the small well-conserved DNA Binding Domain (DBD) that recognizes and targets specific HREs, and (iii) a larger globular carboxy-terminal Ligand Binding Domain (LBD) with the ligand binding pocket (LBP) and the main molecular surfaces involved in dimerization and coactivator recruitment^14^,^15^. The ‘hinge’ is a more variable, unstructured, fragment that connects the DBD to the LBD. Whether the hinge has other specific functional roles remains unclear^16^ and is here explored by our computational analysis. It is clear that NRs are modular in nature and that the isolated domains display intrinsic activities *in vitro*. In addition, there is compelling evidence showing that different domains influence one another in their native environment, adding an extra level of complexity to NR modulation^17^,^18^. For instance, it was recently shown that the dynamics of LBDs is affected by their interaction with DNA^19^. More importantly, ligand-induced activation is dependent on the anchoring DNA sequence^20^,^21^. Although these studies support the existence of synergism and communication between DBDs, LBDs and DNA, significant structural differences between isolated, complexed domains and DNA bound dimers have not been detected that could explain such behavior, suggesting that dynamic or ensemble allostery might play a role in NRs function.

Moreover, in the non-permissive type of heterodimers, heterodimerization and interaction with DNA can impair ligand binding by RXR^22^, suggesting communication between the two subunits. In the permissive type of heterodimers – in which the RXR fully retains its ability to bind ligands – occupancy of RXR’s binding pocket leads to activation of the other NR subunit, either in the absence (transactivation) or in the presence of its own *bona fide* ligand (synergism)^17^,^23^,^24^,^25^, which also supports the existence of functional inter-LBD cooperation.

The latter scenario is common to Peroxisome Proliferator-Activated Receptors (PPARs), which are activated by 9-*cis*-retinoic acid (RTAC) as well as by their own ligands (e.g., fibrate drugs, thiazolidinediones and fatty acid derivatives from diet and metabolism)^8^,^24^,^26^. PPARs are expressed as three subtypes (α, γ, and δ) with distinct although overlapping tissue distributions and impact on metabolism^24^. In particular, subtype γ is a major regulator of glucose and lipid metabolism and has received significant attention due to its implication in modern-life metabolic conditions such as diabetes, obesity, and cardiovascular diseases^27^,^28^.

Structurally, the PPARγ-LBD displays the usual three-layer α-helix fold common to other NRs (Fig. 1), although it differs from others for having an extremely large LBP and an additional helix between helices 2 and 3, named helix 2′^29^,^30^,^31^,^32^. Helix 2′ (H2′) is followed by a lengthy loop commonly referred to as Ω-loop. The Ω-loop and its preceding helix (henceforth referred to as Ω-region) comprise the most conformationally flexile part of the LBD, as revealed by crystallographic B-factors and molecular dynamics simulations^31^,^33^,^34^.

The classic activation mechanism proposes that ligands activate NRs by stabilizing the C-terminal helix (helix 12) in a favorable position to harbor coactivator molecules^16^,^18^,^35^. Such a mechanism is consistent with the observation that PPARγ’s full agonists typically activate the activation function-2 (AF-2) region, interacting with helix 12 (H12) by means of a hydrogen bonding with Tyr473^29^,^30^. However, studies with partial agonists have shown that: i) such ligands do not interact directly with H12^36^,^37^,^38^,^39^,^40^,^41^, and ii) their transcriptional activity is not correlated exclusively with structural or dynamic changes in H12^39^,^41^,^42^. Therefore, other regions of the LBD may play roles in ligand-induced activation, such as the Ω-loop, which appears to be particularly important for PPARγ activation by fatty acids and flavonoids^33^,^43^. An aspect that remains unclear is whether the Ω-loop operates via an allosteric mechanism involving helix 12.

In marked contrast with ligand-induced activation, the allosteric mechanisms by which DNA or RXR ligands modulate PPARγ activity remain poorly understood, partially due to a lack of structural knowledge on how different NR domains are organized in a quaternary structure. Recent breakthroughs, however, have yielded the structures of functional NR complexes bound to DNA^44^,^45^,^46^,^47^,^48^, opening up the possibility of investigating inter-domain communications at the molecular level. Herein, we apply molecular dynamics (MD) simulation methods in conjunction with the community network analysis of correlated motions^6^,^49^,^50^,^51^ to characterize interdependent motions and allosteric pathways responsible for inter-domain communications in the full-length PPARγ-RXRα complex bound to DNA.

## Inter-Domain Correlated Motions

To quantify interdependent motions taking place in the PPARγ-RXRα-DNA complex, we computed inter-residue generalized correlation (GC) coefficients from the MD trajectories. GC analysis produces a matrix of pair-wise correlation coefficients, GC_*ij*_, which measure the extent to which the position (or motion) of residue *i* restricts the range of positions (or motions) available to residue *j*. The resulting correlation matrix of the PPARγ-RXRα complex (Figure S1A) shows that almost all residues in the complex are correlated to one another to some extent (GC > 0.2), reflecting the fact that the residues are all bound – either covalently or by non-bonding contacts – in the same quaternary structure and that large protein regions display rigid body translations. This is especially the case of intra-domain correlations, which arise from rigid body motions of a particular domain within the PPARγ-RXRα/DNA molecular architecture. Since each subunit is formed by a DBD-LBD pair connected by a disordered link (hinge), if one domain translates relative to the others, such motion is expected to produce collective changes of the correlation coefficients for all pairs of residues belonging to that domain. Moreover, the sum of correlation coefficients (see ‘correlation scores’ in Methods) accumulated over that domain would be proportional to the number of its residues. Indeed, we observe such pattern for the internal correlations of residues belonging to domains that undergo translations during the MD trajectories (Figure S2), suggesting that most of the intra-domain correlations in the PPARγ-RXRα complex arise from rigid body-like motions. Because detection of such ‘trivial correlations’ makes it difficult to identify which correlations are truly representative of allosteric mechanisms, we focused our attention on the most intense correlations, which comprise also interdependent conformational transitions in flexible regions.

To filter long-distance and non-trivial correlations, we computed a correlation score per residue that accumulates all intense and inter-domain correlations (CG_ij_^inter^ > 0.6) displayed by each residue *i* (see Methods for details). Figure 2 shows the distribution of such scores along the primary structure (panel A), with the most intense correlations depicted as lines in the three-dimensional structure of the full-length complex (panel B). Interestingly, the most intense inter-domain correlations were not equally distributed along the structure, but rather concentrated in specific regions of the complex that comprise the PPARγ-DBD, conformationally flexible parts (hinges and Ω-loops) and helices that participate in dimerization contacts (H9/10).

Regions that concentrate strong inter-domain correlations are also highly mobile, indicating that they either undergo large conformational changes or execute rigid body like translations within the complex (see RMSF color scale in Fig. 2B). For the PPARγ-DBD, the latter is likely the case, since DBDs are known for being internally rigid domains^52^,^53^. In particular, both the Ω-loops and their preceding helices (helix 2 in RXRα and helix 2′ in PPARγ) show large amplitude correlated motions with one another and with PPARγ-DBD/CTE (C-terminal extension). The RXRα-hinge also appears to be correlated to the motions of the PPARγ-DBD and CTE. In contrast, RXRα-DBD is less mobile and displays only minor correlations with the LBDs.

## Intrinsic dynamics and mobility analysis

We next set out to examine what are the main motions performed by the complex as portrayed by a principal component analysis (PCA). Figure 3 illustrates the dynamics of the complex along the first principal mode of motion (PC1), where the arrows indicate both the direction and the relative amplitude of motions. We found that the dynamics of the hinges and Ω-regions are coupled to the motions of PPARγ-DBD/CTE, which appears to move along with the DNA fragment.

To investigate whether DNA induces such coupled motions, we additionally simulated the PPARγ Hormone Response Element as an isolated DNA stretch (apo-HRE). Conformational analysis revealed that apo-HRE is intrinsically prone to bend, reaching curvatures of up to 50 degrees (histograms in Fig. 4A). When interacting with the NR heterodimer, the bound-HRE has its mobility restricted, particularly in the downstream region, which hosts the RXRα-DBD and on top of which sit the LBDs (bottom panel in Fig. 4A). The upstream region, which hosts the PPARγ-DBD/CTE domain, retains residual motions that are sufficient to promote DNA bending of up to 30 degrees. It is thus likely that the translations displayed by PPARγ-DBD are induced by intrinsic bending motions of the HRE itself. The fact that RXRα-DBD lies on a more constrained region of the HRE is consistent with its reduced mobility as compared to PPARγ-DBD (see Fig. 2).

In contrast, HRE binding has a global effect of enhancing LBD dynamics, as shown by the RMSD distribution of the PPARγ-RXRα-DNA complex (bound-LBD dimer) in comparison to the PPARγ-RXRα dimer (apo-LBD dimer) (histograms in Fig. 4B). Despite such global effect, some particular regions of the heterodimer were stabilized after interaction with the HRE, including PPARγ-H2’ and part of its Ω-loop (bottom panel in Fig. 4B). Therefore, interaction with the HRE appears to induce different effects on each of the LBDs: while it reduces the mobility of PPARγ-H2’, it enhances the motions in RXRα Ω-region. Still, the fact that absolute RMSF of PPARγ-H2’ remains high in the bound state (up to 5 Å in the C-terminal extremity) suggests that the interaction with DNA does not necessarily stabilize it, but rather induces a more coherent type of motion.

## Intra-domain correlations within PPARγ LBD

A generalized correlation analysis was also performed to examine the non-trivial intra-domain correlations within the PPARγ-LBD and their dependence with the LBD oligomerization state (Fig. 5). Comparison of the PPARγ-LBD as a monomer with PPARγ-LBD as part of the full-length complex revealed that most correlations exhibited by the monomer are lost upon heterodimerization and interaction with DNA. However, they are replaced by numerous and strong correlations that do not appear in the monomer and which connect the Ω-region to the helix 12 and its preceding loop (Fig. 5A).

Comparison of intra-domain correlation scores (Fig. 5B) confirms that the native oligomeric state induced a remarkable increase in the accumulated scores of residues lying close to the LBP (Fig. 5C), many of which have either polar or charged side-chains. Interestingly, at least four of these residues have been indicated to be relevant for PPARγ function, according to experimental or theoretical studies (Table 1). A point mutation at I267, located at the Ω-loop, was recently found to abolish PPARγ activation by 15-deoxy-δ^12^,^14^-prostaglandin J_2_^43^. Since I267 has a very prominent role in the correlation network, its functional importance might be related to a key participation in intra-domain allosteric mechanisms, which could be the case of other residues belonging to the Ω-loop. R357 lies at the bottom part of PPARγ-LBD (see Fig. 5C), where it forms salt bridges with E276 and E460, locking the loop 6–7, the bottom part of Ω-loop, and the loop 11–12 together. Computational molecular simulations have suggested that this region comprises an important dissociation pathway by which ligands may escape the ligand binding pocket^54^. Therefore, concerted motions involving R357 might be a mechanism of controlling ligand dissociation kinetics. Two other residues whose correlations emerged in the native oligomeric state lie at helix 12 and are engaged in important hydrophobic contacts (P467) or electrostatic interactions (E471) that hold the coactivator peptide in place^29^,^55^. Allosteric modulation of these residues by distant regions such as the Ω-loop can thus directly impact PPARγ ability to recruit coactivator proteins.

## Residual Local Frustration

To quantify conformational residual local frustration in the crystallographic structure of the full-length complex, we applied the frustratometer algorithm^56^. The frustratometer measures how ‘frustrated’ a residue is by checking how conformational/mutational changes in its vicinity shift the residue energetics^13^. According to the energy landscape theory, while minimally frustrated contacts are important to stabilize the folded core of the protein, local clusters of highly frustrated contacts might have evolved so that proteins can easily adapt their structure and modulate their function in response to their environment^13^.

Figure 6 shows the distribution of minimally (green) and highly (red) frustrated contacts along the quaternary fold. As expected, DBDs along with the inner core of LBDs and dimerization regions are dense with minimally frustrated contacts. The PPARγ-hinge, in contrast, was found to be the most frustrated region of the complex, suggesting that it works as a ‘molecular spring’ that can change shape in response to DNA distortions^48^. Highly frustrated contacts were also observed in the RXRα-hinge, both the Ω-regions and other helices and loops near the proteins’ surface (H1, H2, loop 3–4, H6 to H7, loop 11–12, and H12). Interestingly, some of the highly frustrated clusters map well to highly correlated regions (compare to Fig. 2), reinforcing that frustrated regions might have additionally evolved to undergo interdependent conformational changes. Since flexible regions are separated by minimally frustrated contacts at the folded core of the complex, in the next section we investigate how information is transmitted along the structure through interdependent motions.

## Weighted protein network and preferential allosteric pathways

To compute the relative contribution of different types of residues to the overall correlation network, we devised a ‘*contribution score*’, computed for each residue type *i* as the difference between the *accumulated score* (AS*i*) and the *expected score* (ES*i*), where AS*i* is the sum of the correlation coefficients involving at least one residue of type *i* and ES*i* is computed as:


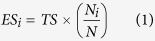


where TS is the sum of all generalized coefficients in the complex, *total score*, N*i* is the number of residues of type *i* and N is the total number of residues in the complex. Therefore, the ES*i* depends only on the number of occurrences of each residue type in the structure. It reflects the relative contribution that would be expected if every type of amino acid contributed with the same intensity to the overall network of correlations, irrespective of the side-chain nature. By subtracting the ES*i* from the observed AS*i*, we obtain a metric of the deviations from this behavior, which we call *contribution score*. While positive values reflect a more important contribution than would be expected if only the backbone atoms mattered for conveying information, negative values reflect an unexpectedly low contribution to the overall network.

It is evident from Fig. 7 that aliphatic and apolar amino acids tend to contribute less to the allosteric communication than would be expected from their population in the primary sequence. On the other hand, arginines display an unexpected high contribution, followed by histidines and, to a lesser extent, glutamines and asparagines. Such intense participation in allosteric transmission could be either related to their location in the quaternary fold or to the very nature of their polar side chains.

We also used a weighted protein-network approach to identify the ‘shortest’ pathways connecting highly correlated residues that lie far apart in the three-dimensional structure. In this approach, the whole structure is represented by a set of nodes (i.e. residues) connected by edges whose *length* (w_*ij*_) depends on the GC coefficients (see Methods for more details). Based on such representation, the shortest pathways are those that simultaneously minimize the spatial distance (i.e. the number of nodes involved in the pathway) and maximize the allosteric efficiency (i.e. the intensity of the GC coefficients along the pathway).

This approach was applied to find the preferential pathways connecting the Ω-regions and PPARγ-DBD/CTE. Interestingly, we found that the ‘shortest’ allosteric pathways avoid the more rigid core of the complex by going through solvent exposed loops and helices and by making use of highly frustrated contacts (Fig. 8, left panels). The PPARγ-hinge, despite being highly frustrated, does not comprise any important allosteric pathway. Therefore, DNA bending motions appear to translate into distant conformational changes by means of PPARγ-DBD translations, not via the PPARγ-hinge.

Additionally, while communication within the same or even neighboring secondary structures are usually degenerated, distinct secondary structures communicate by means of key interactions that generally involve at least one polar or charged residue (Fig. 8, right panels). This is particularly evident in case of communications between the two NR subunits, which take advantage of hydrogen bonds connecting i) PPARγ Q451 (H11) to RXRα E434 (H11), ii) RXRα K395 (H9) to PPARγ Q430 (L9-10), and iii) RXRα E217 (hinge) to PPARγ N151 (DBD).

## Community Network and Vertex Betweenness

We have applied a community network analysis^6^,^49^,^50^ to identify groups (or ‘communities’) of residues that are closely correlated. Such communities are correlated with each other through key amino acid residues that establish contacts critical for long-range allosteric mechanisms^6^.

The resulting communities obtained from our network analysis of MD simulations are depicted in Fig. 9A. The weighted protein-network is divided into communities based on the flow of allosteric information that passes through each pair of nodes (or ‘edge’). This is measured by the edge betweenness parameter, defined as the number of shortest pathways that pass through an edge. By looking at the residue pairs with the highest edge betweenness, we could also identify the strongest and best conserved contacts involved in inter-community communications, displayed in Fig. 9B.

**Figure 1:**
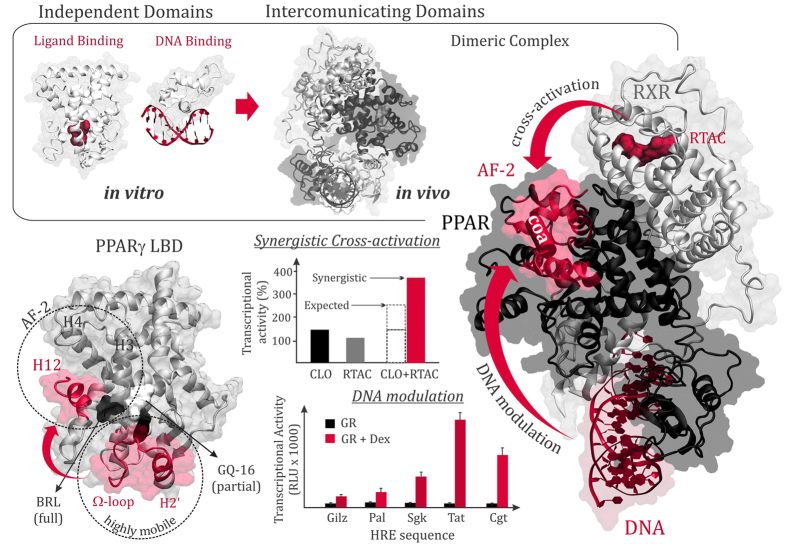
Allosteric communications in the PPARγ-RXR α nuclear receptor (NR). NRs are modular proteins formed by ligand (LBD) and DNA (DBD) binding domains that display intrinsic activity *in vitro*. *In vivo*, they form dimeric complexes, such as the PPARγ-RXRα complex, that bind to DNA and show allosteric interdomains communication (top and right panels). RXR ligands, such as 9-*cis*-retinoic acid (RTAC), can cross-activate PPAR by inducing conformational changes in its activation function-2 (AF-2) region, comprising the helix 12 (H12) in the LBD. Synergistic cross-activation is known to take place when PPAR is bound to one of its own *bona fide* ligands, such as clofibric acid (CLO, illustrative histograms reproduced from ref. 23). As observed for other NRs (such as glucocorticoid, estrogen and vitamin D receptors), transcriptional activity can be modulated by the DNA sequence (illustrative histograms reproduced from ref. 21), suggesting allosteric communications between the DNA and the AF-2 region. The PPARγ LBD (bottom left panel) differs from other NRs by having an extra helix (H2’) followed by an extremely large and flexible Ω-loop. Full agonists, such as rosiglitazone (BRL), activate the AF-2 region by forming a direct interaction with the H12, while partial agonists (such as GQ-16) do not make direct contact with H12, indicating alternative allosteric mechanisms that may involve the Ω-loop and H2’ flexible regions.

**Figure 2:**
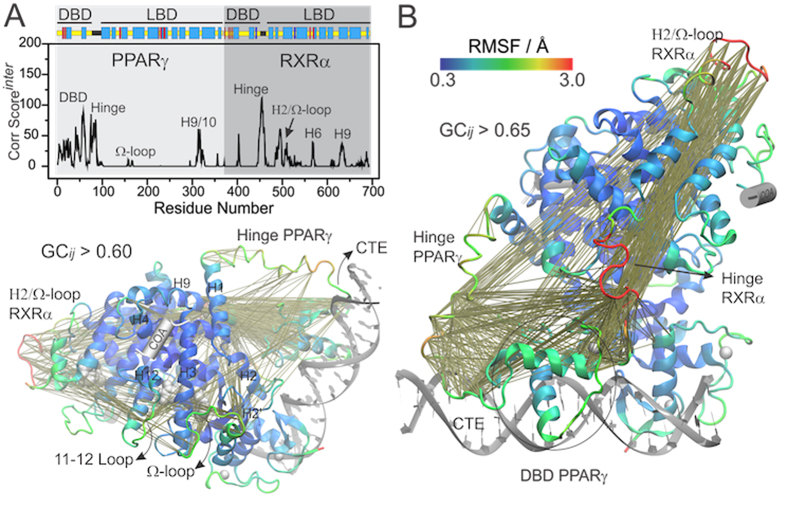
Large-scale inter-domain correlated motions on the full-length complex. (**A**) Correlation score per residue, computed for inter-domain correlations with GC_*ij*_^*inter*^ > 0.6. (**B**) Localization of the most intense inter-domain correlations in the three-dimensional structure. The heterodimer PPARγ-RXRα was colored according to backbone RMSF, while other components are represented in silver. For the sake of clarity, the bottom left panel only shows inter-domain correlations that involve PPARγ residues.

**Figure 3:**
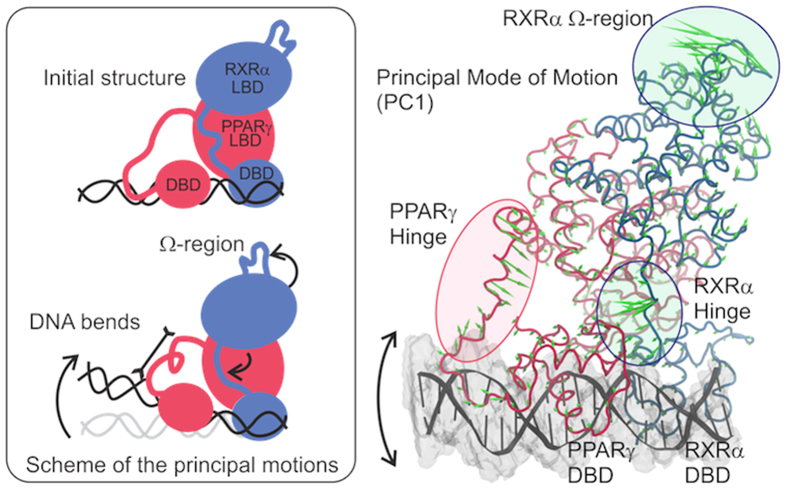
Principal mode of motion (PC1) reveals translations of PPARγ-DBD and coupled conformational changes in the hinges and Ω-regions. PC1 is represented schematically (left panel) or by arrows of sizes proportional to the amplitude of motion (right panel). DNA occupancy map is shown as a grey surface in the right panel.

**Figure 4:**
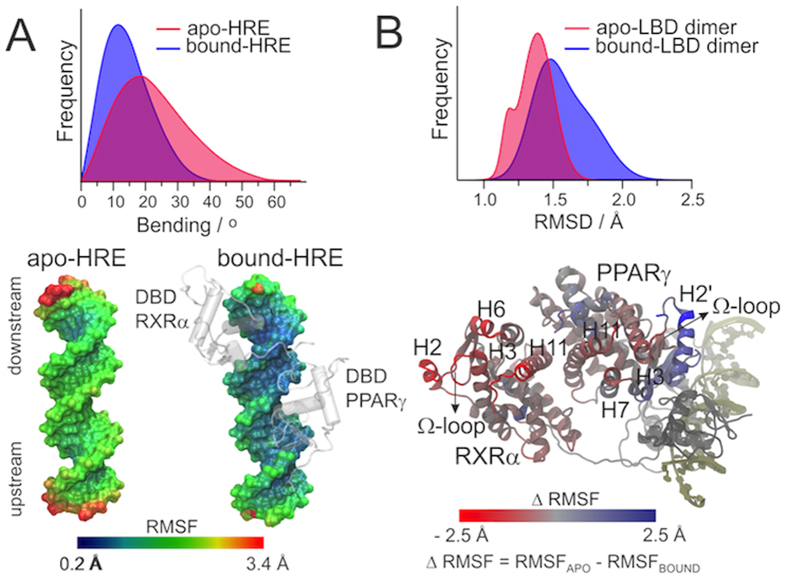
Mobility analysis comparing apo- or bound- states of the HRE (A) and the LBD dimer (B). The HRE mobility is shown in terms of bending (histograms) and RMSF (bottom panel). For the sake of clarity, only the DBDs are shown in the bound-HRE state. The LBD dimer mobility is shown in terms of RMSD distribution (histograms) and differential RMSF (bottom panel). Differential RMSF was computed by subtracting the RMSF in the bound state from the RMSF in the apo state.

We found that DBDs behave as individual communities (communities 0 and 4 in Fig. 9A), while LBDs display some dissociation between their ‘upper’ (communities 2 and 5 for PPARγ and RXRα, respectively) and ‘lower’ halves (communities 3 and 6, for PPARγ and RXRα, respectively), which appear as distinct though strongly connected communities. (The ‘upper’ and ‘lower’ parts are defined relative to the LBD orientation used in Fig. 1). Such community structure is consistent with NR’s modular nature, since DBDs, lower- and upper-LBDs have distinct and well-defined functions: DNA binding, ligand binding, and dimerization, respectively. Interestingly, the connection between upper-LBDs (communities 2 and 5) is stronger than the connections between upper and lower parts within each LBD (communities 2 and 3 for PPARγ and communities 5 and 6 for RXRα), which highlights how effective the dimerization contacts are. Dimerization contacts also allow for communication between the two ligand binding pockets, as indicated by the sizeable interconnectivity of the lower LBD communities (connection between communities 3 and 6), with information flowing through the PPARγ-Y477/RXRα-E434 H-bond interaction connecting the two H11 helices (see Fig. 9B, bottom panel). Interestingly, the PPARγ-hinge appears as an extremely isolated community (community 1), displaying only weak communications with PPARγ DBD and LBD. This is consistent with the fact that the PPARγ-hinge appears as the most frustrated region in the complex. The RXRα-hinge, on the other hand, takes part in two communities that are mainly formed by PPARγ-DBD and RXRα-LBD.

Edge betweenness analysis revealed a strong flow of allosteric information between PPARγ-DBD, PPARγ-LBD, and RXRα-hinge (Fig. 9B, top panel). A closer inspection reveals that it involves mainly electrostatic interactions between i) PPARγ Arg153 or His155 (DBD) and PPARγ Glu427 (loop 9–10) and ii) PPARγ Ser429 (loop 9–10) and RXRα Glu395 (hinge). Again, medium/long-chain charged or polar residues appear to be fundamental in conveying allosteric information between different subunits. In particular, PPARγ 9–10 loop appears to form an important allosteric center mediating communication between PPARγ-DBD and both LBDs. Another important edge is formed by PPARγ Tyr477 (H12) and RXRα Glu434 (H11) (Fig. 9B, bottom panel). Analyses of the MD trajectories confirmed the existence of solvent-mediated hydrogen bond interactions linking Glu434 side-chain to Tyr477 backbone. Such residues displaying high betweenness are likely to play important roles in allosteric mechanisms and should be good candidates for mutagenesis studies.

## Discussion

In recent years, multidomain structures of NR complexes provided us with the first insights on how quaternary architecture influences NR function^44^,^45^,^46^,^47^,^48^. However, only a few structures are available, which capture the average conformation adopted by these complexes and, hence, information on the role of dynamics for allosteric mechanisms in physiological conditions is still rather limited. Here we report on the dynamics of the first X-ray structure of a full-length NR complex by means of molecular dynamics simulations. We used a combination of generalized correlation coefficients, principal component analysis (essential dynamics), residual local frustration analysis, and community networks to investigate correlated motions that enable distant regions of PPARγ-RXRα-DNA complex to allosterically communicate.

Generalized correlation analysis revealed that distant mobile regions of the complex undergo large-scale interdependent motions. Altogether, conformationally flexible parts of both LBDs appear to have their dynamics connected to PPARγ-DBD/CTE, but not to RXRα-DBD. Importantly, these mobile regions encompass the Ω-loops and their preceding helices (Ω-regions), which are strongly correlated to PPARγ-DBD and its C-terminal extension, inserted in the DNA minor groove. The Ω-loops comprise the most flexible parts of the LBDs and have been recently suggested to be important modulators of PPAR function in addition to or in combination with H12^33^,^34^,^43^. Therefore, our findings suggest that the Ω-loops could work as important molecular switches that modulate PPARγ function in response to the dynamics of PPARγ-DBD.

Essential dynamics of the full-length complex confirmed that PPARγ-DBD and its CTE undergo rigid-body translations relative to the LBDs. This is in agreement with recent crystallographic data on the RXRα-LXRb complex showing significant differences in the relative positions of the DBDs within the asymmetric unit of the crystal^48^. In our simulations, such translations appear to be induced by the HRE, which is intrinsically prone to bending. Although the comparative analysis of the mobility indicated that the HRE loses motional freedom upon interaction with the full-length complex, the NR-bound HRE retains residual bending motions that are sufficiently large to translate the PPARγ-DBD/CTE domain and also to induce and control the dynamics of distant regions of the LBD dimer, including the Ω-regions. Thus, consistent with the hypothesis of DNA being an active player on transcription^1^,^20^,^21^, our simulations suggest there is a flux of conformational information flowing from DNA to the LBDs, which is mediated by the PPARγ-DBD. Since small differences within a DNA sequence or in its flanking regions can produce large effects on its intrinsic flexibility, HRE dynamics – not its average conformation – could be the hidden information that is interpreted by bound NRs and translated into different levels of transcriptional activity, as previously reported^20^,^21^.

The RXRα-DBD, in turn, appears to have a much less prominent role than PPARγ-DBD on reading DNA dynamics, probably owing to its placement in a more constrained region of the HRE. Such observation is consistent with a recent Hydrogen/Deuterium Exchange study on the VDR-RXR complex, which has shown that RXR-DBD dynamics is less affected by DNA binding as compared to its partner DBD^19^. Despite the fact that the RXRα-DBD contribution for allosteric communication is likely to differ in other quaternary architectures, in PPARγ-RXRα complex its role appears to be limited to anchoring the complex to DNA, while the PPARγ-DBD works both on anchoring and allowing sequence-specific motions of the HRE to affect the dynamics of the two LBDs and, possibly, their function.

Within PPARγ-LBD, motions appear to be strongly dependent on the native oligomeric state, which queue the Ω-region and H12 to move concertedly. Such coherence could be enforced either by i) steric constraint imposed by other domains of the complex, ii) DNA-induced motions or iii) a combination of both. At any rate, the native oligomeric environment appears to consolidate an allosteric mechanism by which the conformation and dynamics of the Ω-loop could modulate H12 function, consistently with previous reports^34^,^43^. Our correlation analysis suggests that the intra-domain correlation network is mainly formed by residues surrounding the LBP, some of which have been suggested or proven to affect PPARγ function. While some of these residues directly interact with coactivator peptides (Pro467^55^ and Glu471^29^), it is less evident how Ile267^43^ or Arg357^54^, which are removed from H12, could modulate PPARγ activity. We suggest that their functional importance is strongly related to their participation in intra-domain allosteric mechanisms, by which they could influence H12 dynamics (Ile267) or ligand dissociation kinetics (Arg357). Since PPARγ depends both on dimerization with RXRα and on binding to DNA to be transcriptionally active, other residues whose correlations increase in the native state are likely important for function and may be good candidates for novel mutagenesis studies.

Remarkably, strongly correlated regions such as the Ω-regions and PPARγ-DBD/CTE are not spatially proximal, hinting at how complex is the mechanism by which allosteric information travels along the heterodimer. Moreover, in the space between such highly correlated regions lies the folded core of the complex, formed by minimally frustrated residues. While highly frustrated residues display unfavorable contacts that allow for large amplitude motions without significant energy cost, minimally frustrated residues are so comfortably placed that even small scale motions are energetically expensive^13^. Therefore, it would be naïve to assume that communication between distant and flexible regions occurs by a single (or even a few) allosteric pathway, which would require large-scale motions in the minimally frustrated core. Instead, we propose that communication between largely flexible regions must arise as a cooperative effect emerging from an extensive and complex network of small/medium amplitude motions throughout the structure.

This is equivalent to saying that the system’s conformational energy landscape has been shaped to have multidimensional canyons as shown in Fig. 10. Therefore, some of the highly frustrated regions in the complex appear to have evolved to display interdependent motions, as previously proposed by Ferreira *et al*.^57^. Considering the large number of global conformations that would be available if the motions of two highly frustrated regions were completely independent of each other, such canyon-like topography might have evolved to restrict unproductive combinations of local conformations and, thus, accelerate transitions between functionally active states in proteins and other macromolecules.

We also found that polar amino acids have a more prominent role in the correlation network than would be expected from their frequency in the primary structure, thus suggesting the existence of preferential allosteric pathways by means of electrostatic interactions. This is particularly true for arginines, which is consistent with their recently described role as the most effective heat diffusers in PPARγ and in other proteins^58^. Weighted protein-network analysis also revealed that while internal correlations tend to be distributed in several degenerated pathways, they funnel down into key electrostatic and polar interactions when it comes to inter-domain communication.

As a general trend, the shortest correlation pathways connecting strongly correlated regions exploit frustrated regions at the surface of the complex, avoiding the minimally frustrated core. They comprise medium- or long-chain polar and charged residues near the surface of the proteins, especially at the PPARγ 9–10 loop. This region represents a ‘convergence zone’, connecting the PPARγ DBD to both LBDs. A similar ‘convergence center’ was recently reported for the HNF-4α NR complex, where single-residue modifications were found to significantly alter DNA binding and NR transcriptional activity^45^. Arg91, located at the upstream DBD, protrudes into the HNF4-α convergence center, similarly to what was observed for Arg153 in the DBD of PPARγ. In PPARγ, Arg153 protrudes towards loop 9–10, forming a salt bridge that was found to have a very strong betweenness in the weighted network. Additionally, the strong interconnectivity between RXRα Glu434 (H11) and PPARγ Tyr477 (12), mediated by solvent molecules, could be important for the asymmetric permissiveness observed in the PPARγ-RXRα heterodimers, by which RXRα ligands can activate PPARγ^25^. Our results thus support the idea of important ‘convergence centers’ for propagating allosteric information, as in the compact folds reported by Chandra *et al*.^44^,^45^. This interpretation is apparently inconsistent with an alternative “open” architecture for the intact complex proposed on the basis of low-resolution structural methods^59^. A thorough discussion about the architecture of PPAR-RXR and other full-length NRs is found in Rastinejad *el al*.^60^.

The upper half of the PPARγ LBD appeared as the central community in the complex network, consistently with its central position in the asymmetric quaternary organization reported by Chandra *et al*.^44^. While the RXRα-hinge appeared as part of the PPARγ-DBD community, PPARγ-hinge appeared as an independent and poorly connected community. It is thus likely that PPARγ-hinge has evolved as a highly frustrated region that easily adapts its length to buffer the impact of DNA bending modes on the heterodimer stability. This view stands in contrast with previous hypotheses of hinges working as ‘rigid spacers’^46^,^47^ that have been more recently denied^60^, and in agreement with the suggestion that the RXRα-hinge plays an important functional role modulating the relative distance between DBDs and LBDs in the RXRα-LXRβ complex^48^.

## Conclusions

We have elucidated the allosteric pathways in DNA-nuclear receptor (NR) complexes, based on the first X-ray structure of a full-length DNA/NR complex. The analysis of correlated motions in the DNA/PPARγ-RXRα complex and in the isolated PPARγ-LBD monomer reveals highly correlated motions between distant mobile regions of the complex, with the most prominent inter-domain correlations found at the conformationally flexible hinges, Ω-loops and helices that participate in dimerization contacts (H9/10) of PPARγ-DBD. We found that the oligomerization state affects the correlated motions within the PPARγ-LBD. The Ω-region motions involved in inter-domain correlations also affect those of helix-12, i.e. the target of PPARγ’s full agonists. Remarkably, such concerted motions are absent in the PPARγ-LBD monomer.

The reported analysis provides a rigorous interpretation of experimental observations from mutagenesis studies and suggests other amino acid residues expected to be crucial for PPARγ’s transcriptional activities. The principal component analysis of MD trajectories supports the central role played by highly correlated regions during allosteric modulation of DNA/NR complexes. In fact, the analysis of essential dynamics indicates that, upon formation of the NR-DNA complex, residual DNA motions (with bending angle up to 30 degrees) translate the PPARγ-DBD/CTE domain and affect the dynamics of distant regions, including the Ω-loop in the LBD dimers. In response to DNA distortions, NR hinges change in shape, functioning as ‘molecular springs’, while the RXRα-DBD remains relatively rigid and displays only minor correlations with the LBDs. This suggests that the RXRα-DBD anchor the NR heterodimer to the DNA, while the flux of conformational information between DNA and the LBD domains is established through the PPARγ-DBD. These results support the hypothesis that DNA dynamics (with specific promoter sequence) play an active role in transcription activities.

Our residual frustration analysis revealed that highly correlated regions map well with some highly (mobile) frustrated clusters, indicating that flexible regions could undergo interdependent conformational changes associated with allostery. However, these flexible regions are physically separated by minimally frustrated contacts at the folded core of the complex, whose motions are energetically expensive. Our weighted network, based on mutual information of correlated protein motions, provides clear pathways for communication between distant flexible regions. We find evidence that allostery avoids the more rigid core of the complex and makes use of highly frustrated contacts, passing through solvent exposed loops and helices. Our community network analysis also shows that distinct secondary structures communicate through polar amino acid residues, with convergence centers linking different subunits of the NR complex, such as PPARγ 9–10 loop and RXRα(H11)-PPARγ(H12). Notably, we found that the PPARγ-hinge, despite being the most frustrated region of the complex, it is not involved in any important allosteric pathway and consequently remains as an isolated community in the complex network.

We conclude that sequence-dependent DNA bending motions are correlated with the ligand binding domains of the nuclear receptor by translation of one DNA binding domain (PPARγ-DBD). Allosteric pathways involve highly frustrated clusters and specific polar amino acid residues. Future experiments might provide further characterization supporting the reported insights on allosteric pathways in DNA-NR complexes, based on the generalized correlation analysis, essential dynamics, residual local frustration analysis, protein network theory and community network analysis. These methodologies already provide valuable insights on structure/function relations sites that could lead to new strategies in the development of allosteric drugs that target protein-DNA complexes, especially considering that no such drugs have yet been uncovered to date^16^,^44^. These methods are expected to be useful, in general, for other systems where allosteric processes are critical, including allostery in prokaryotic immune systems with regularly interspaced short repetitions of base sequence (CRISPR-Cas9), currently emerging as a revolutionary tool in structural and molecular biology^61^.

## Methods

### Molecular Dynamics Simulations

Four different systems were simulated: full-length complex, PPARγ-RXRα LBD heterodimer, PPARγ-LBD as a monomer and apo-HRE. The initial coordinates were taken from crystallographic structures deposited in the Protein Data Bank with the corresponding entries: 3ZDY (full-length complex)^44^ and 1FM6 (heterodimer and monomer)^32^. The initial structure of apo-HRE was also taken from PDB 3ZDY after removal of the non-nucleic parts. The Ω-loops (residues 242 to 265 in PPARγ and 260 to 275 in RXRα) – which were missing in the full-length structure – were taken from 1FM6 after an alignment of the structures had been performed with LovoAlign^62^. Hydrogen atoms were added and protonation states of ionizable groups were estimated using the server H++^63^ at pH 7.

Complete simulation boxes were built with VMD^64^. Structures were solvated with TIP3P water molecules^65^ within rectangular boxes such that the solute molecules (protein, DNA and ligands) were at least 20 Å from the box boundaries. Sodium and chloride ions were added in order to make the systems electrically neutral at physiological concentration (0.154 mol/L). The ions were placed at favorable positions as determined by electrostatic potential calculations with MeadIonize VMD plugin. Complete systems consisted of approximately 160,000 atoms (full-length complex), 85,000 atoms (LBD heterodimer), 60,000 atoms (PPARγ-LBD monomer) and 40,000 (apo-HRE).

Simulations were performed with NAMD by applying periodic boundary conditions and an integration time-step of 2.0 fs. All bonds involving non-polar hydrogens were constrained at their equilibrium length using the SETTLE algorithm, as implemented in the NAMD code^66^. Pressure (1 atm) and temperature (310 K) were controlled using the Langevin/Nosé-Hoover barostat and the Langevin thermostat, respectively.

CHARMM parameters^67^ were applied for protein, DNA and 9-*cis*-Retinoic Acid, while Rosiglitazone parameters were obtained from Hansson *et al*.^68^. A 12 Å cutoff with smooth switching function starting at 10 Å was used for van der Waals interactions and Particle Mesh Ewald method was applied to evaluate electrostatic interactions, as implemented in NAMD.

Minimization and equilibration procedures consisted of i) 2000 gradient conjugate minimization steps and ii) 200 ps of molecular dynamics simulations, keeping the solute molecules fixed (proteins, DNA and ligands). Thereafter, these procedures were repeated keeping only the protein α-carbons and the DNA backbone fixed. As a last equilibration step, a 2 ns simulation without restraints was performed. After these procedures, 3 independent production trajectories were generated for each system (full-length complex, PPARγ-RXRα LBD heterodimer, PPARγ-LBD as a monomer and apo-HRE), lasting 40 ns each. Here, we assume that the motions in the first 40 ns are those crucial for determining the allosteric mechanism. This assumption is corroborated by the converge study reported in the Supporting Information (Fig. S1), based on longer MD simulations involving: i) simulation time extension of the three original trajectories from 40 ns to 120 ns; and ii) addition of other three independent simulations of 100 ns; for a total simulation time of 0.66 microseconds.

### Mobility and conformational analysis

Protein mobility analyses (RMSD and RMSF) were performed with a home-made analysis suite. DNA conformational analysis was performed with an adapted version of program CURVES + 69. DNA bending histograms were generated with Canal^69^. Trajectory visualization and pictures were made with VMD^64^.

### Generalized Correlation Analysis

For the analysis of correlated motions, we used the method of generalized correlations (GC), proposed by Lange *et al*.^70^. In comparison with the more traditional Pearson coefficients, GC analysis has the advantages of i) being independent of the relative orientation of the atomic fluctuations and ii) being able to capture non-linear correlations. GC analysis is based on the statistical concept stating that two random variables can only be considered independent if their joint probability distribution, *p*(x_*i*_, x_*j*_), equals the product of their marginal distributions, *p*(x_*i*_).*p*(x_*j*_). If the values adopted by x_*i*_ somehow restrict the range of values accessible to x_*j*_, then the joint probability is smaller than *p*(x_*i*_).*p*(x_*j*_). Such deviation thus reflects the degree of correlation between x_*i*_ and x_j_ and is called mutual information (MI), defined as:


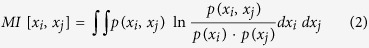


MI is closely related to the concept of Shannon entropy (Equation 3), which states that the expected information content of a discrete random variable x, having a probability distribution *p*(x) corresponds to:


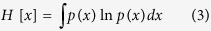


Therefore, the mutual information associated to random variables x_*i*_ and x_j_ can be computed as in Equation 4, where *H*[x_*i*_] and *H*[x_*j*_] correspond to marginal entropies, and *H*[x_*i*_, x_*j*_] corresponds to the joint entropy, which can be estimated with different methods. In this work, generalized correlations were computed with the g_correlation tool^70^ built to work within GROMACS 3.3^71^. The g_correlation tool estimates marginal (*H*[x_*i*_] and *H*[x_*j*_]) and joint (*H*[x_*i*_, x_*j*_]) entropies by means of the *k*-nearest neighbor distances algorithm^72^ applied to the atomic positions fluctuations from MD simulations.


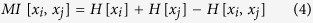


Because MI varies from 0 to +∞, the GC coefficients defined as in Equation 5 provide more intuitive values ranging from 0 (independent variables) to 1 (fully correlated variables)


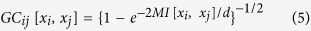


where *d* is the dimensionality of the variable x.

A complete convergence study of the correlated motions in the 40 ns time window and a comparison with longer MD simulations is provided in the Supplementary Information (Fig. S1).

### Correlation Score Function

GC coefficients were also used to build a correlation score function that is a measure of both the number and the intensity of correlations displayed by each residue, defined as:


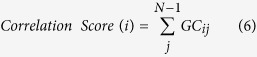


By tracking the residues that display large correlation scores, such function helps to point out the most important regions to orchestrate correlated motions. The correlation score function can be filtered to include only intra- or inter-domain correlations. Additionally, an *intensity cutoff* parameter can be used to filter only the most intense correlations. Once computed for every residue in the structure, the correlation score can also be accumulated over a group of residues belonging to the same domain or displaying the same side chains.

### Principal Component Analysis and Free Energy Landscapes

Essential motions of the system were obtained by Principal Component Analysis (PCA). PCA consists in diagonalizing a variance-covariance matrix of the system 3 N atomic positional fluctuations in order to obtain a new set of coordinates (eigenvectors) to describe the system motions. PCA allows decomposing the total motion described by the system into several independent (orthogonal) motions of varying time scales and amplitudes. Each eigenvector (or principal component, PC) has an associated eigenvalue corresponding to the mean square fluctuation contained in the system’s trajectory projected along that eigenvector. By sorting the eigenvectors according to their eigenvalues, the first principal component (PC1) corresponds to the system’s highest amplitude motion, generally approximated as its ‘essential dynamics’. In this work, PCA was performed with a home-made program.

**Figure 5:**
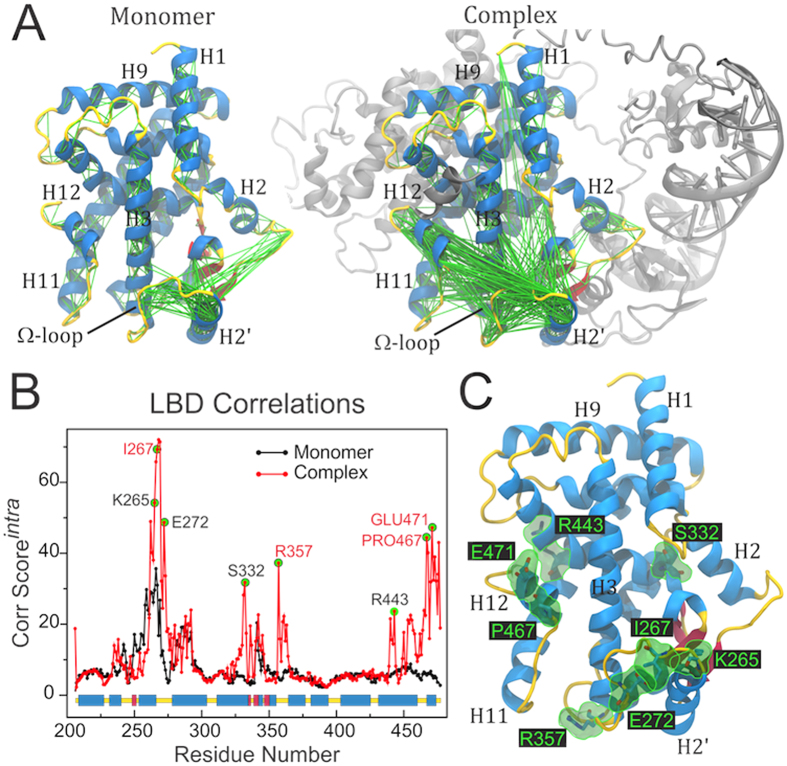
Correlation network within PPARγ-LBD depends on the oligomeric state. (**A**) Intense correlations (GC_*ij*_^*intra*^ > 0.6) displayed by PPARγ-LBD as a monomer (left) or as part of the full-length complex (right). (**B**) Correlation score per residue, computed for GC_*ij*_^*intra*^ > 0.6. Residues written in red are known to be important for function. (**C**) Localization of some of the most intensely correlated residues on the LBD structure (marked in green in (**B**)).

**Figure 6:**
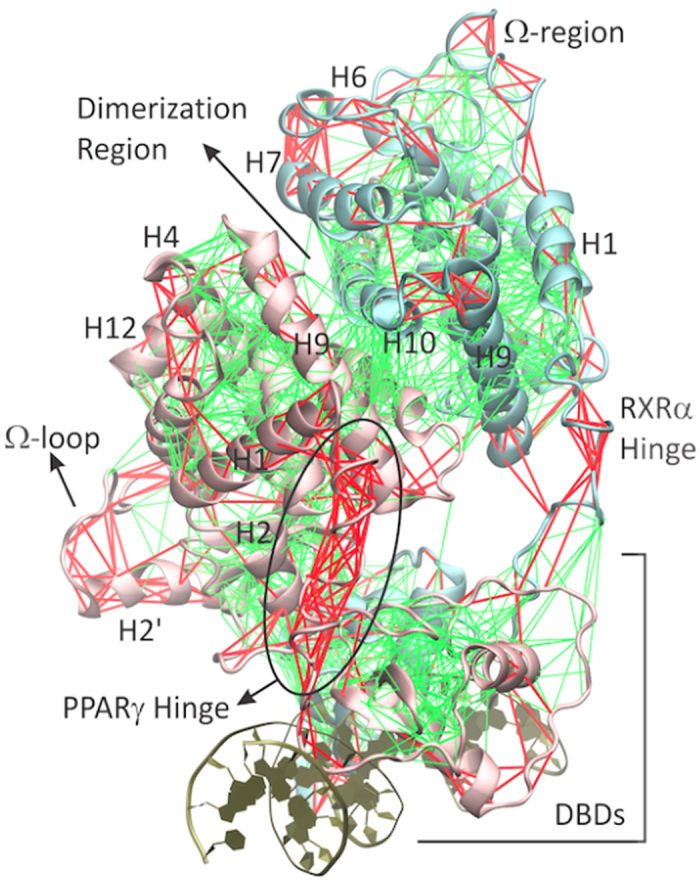
Conformational residual local frustration analysis. Minimally frustrated contacts (green lines) are concentrated in the DBDs, the folded core of the LBDs, and the hetero-dimerization surface. Highly frustrated contacts (red lines) are clustered in the hinges, Ω -loops, and in surface helices and loops.

**Figure 7:**
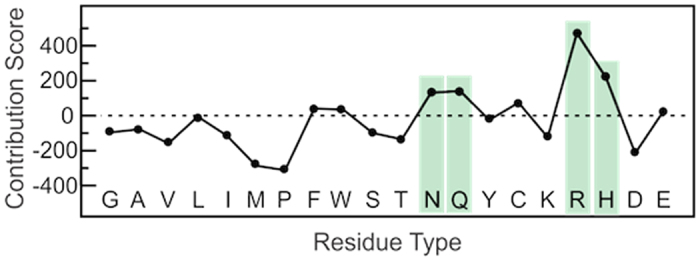
Contribution Score computed for each residue type. Positive (or negative) values reflect residue types that contribute more (or less) to the correlation network than would be expected from their population in the primary sequence.

PCA analyses showed in Figs 5 and 10 have been performed for a single MD trajectory of 40 ns. To generate the energy landscapes shown in Fig. 10, we performed independent PCA analysis on selected regions of the complex and computed the corresponding free-energy surfaces according to Equation 7, where k_*B*_ is Boltzmann constant, *T* is the temperature, *P*(q) is an estimate of the probability along the variable q and *P*_*MAX*_(q) is the probability of the most probable state. *P*(q) and *P*_*MAX*_(q) were obtained from histograms of the MD trajectories projected along PC1.


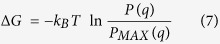


### Residual Local Frustration Analysis

Residual Local Frustration is essentially a measure of how ‘comfortable’ a residue is in terms of the energetics of interaction with its neighbor residues in the native structure of a protein or macromolecular complex^13^. To quantify local frustration in the PPARγ-RXRα complex, we submitted the pdb structure (3DZY) to the frustratometer web server (http://www.frustratometer.tk/). The frustratometer algorithm quantifies local frustration by applying conformational changes (or mutations) in the structure and analyzing how such changes affect the interaction energies. Residues that are systematically destabilized by random changes in their vicinities are considered minimally frustrated, while those that are systematically stabilized are considered highly frustrated^56^.

### Weighted Protein-Network, Shortest Pathways and Community Analyses

The communication network is defined as a set of nodes (amino acid residues), connected by edges (residue pair connections) whose length is weighted using the generalized correlation coefficients, GC. Two nodes are considered connected if the heavy atoms are within a *distance cutoff* (5.5 Å) for at least a *frame cutoff* (75% of the MD frames analyzed). The choice of the cutoff parameters is justified at the end of this section. The edge lengths in the ‘weighted protein-network’ are calculated as w_*ij*_ = −log(GC_*ij*_), with GC_*ij*_ defined as in Equation 5. Such a weighted graph defines the dynamical network that contains information paths and critical edges/nodes that are crucial for communication within the complex. As indicated in the “Generalized Correlation Analysis” paragraph, the generalized correlation coefficients have been obtained as averages of three independent 40 ns simulations.

**Figure 8:**
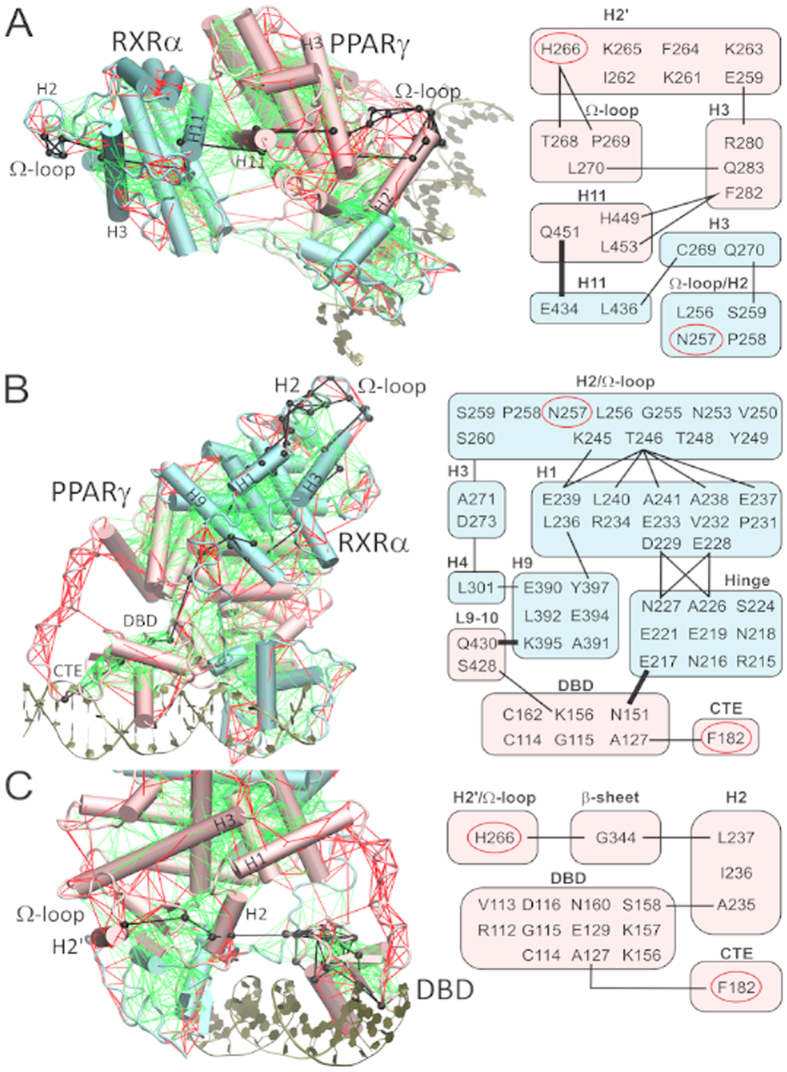
Shortest pathways connecting (**A**) RXRα Ω-loop to PPARγ Ω-loop, (**B**) RXRα Ω-loop to PPARγ -CTE and (**C**) PPARγ Ω-loop to PPARγ-CTE. Left panels indicate the shortest pathways as black lines in the three-dimensional structure, along with highly (red) and minimally (green) frustrated contacts from residual frustration analysis. Right panels schematize the shortest pathways connecting different secondary structures, with inter-domain communications highlighted in bold.

Within the weighted protein-graph, the ‘shortest pathways’ connecting any pair of residues have been calculated using the Floyd-Warshall algorithm^73^. This algorithm sums up the *lengths* (w_*ij*_) of all edges involved in different paths of nodes connecting two distant residues and identifies the pathway displaying the shortest total length. For specific residue pairs showing large long-range correlations (see Fig. 8) we have calculated all possible pathways (not only the shortest) within the weighted protein-network. The communication pathways could be very close in length to the shortest pathway (sub-optimal pathways), where the pathway length is defined as the sum of the edge lengths involved in that pathway. This likely happens when the shortest pathways involve highly correlated residues within the same or nearby secondary structures. For simplicity, we consider as sub-optimal pathways only those whose lengths are not larger than 2% of the shortest pathway length.

In the weighted network, there are groups of residues (‘communities’) within which the connections are dense but between which they are sparse. These local substructures can be obtained using the Girvan-Newman algorithm^74^, a divisive algorithm that is essentially based on the use of the *edge betweenness* as partitioning criterion. The edge betweenness measures signal traveling through a network and is defined as the number of shortest pathways that cross an edge^74^, providing a parameter that favors edges that interconnect communities and disfavors edges that lie within communities. The edges with the highest betweenness connect many pairs of nodes and form the link between different communities. High edge betweenness also associate with pairs of residues that are important for the communication flow within the protein-network.

**Figure 9:**
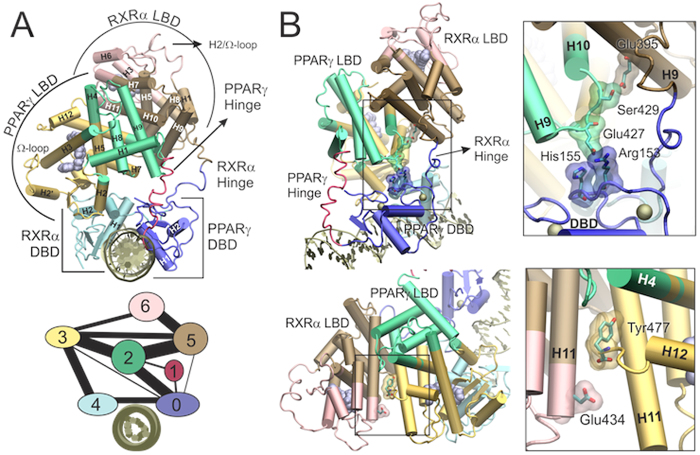
Community network analysis. (**A**) Community structure of the full-length complex displayed in the three-dimensional structure (top panel) or in schematic two-dimensional representation (bottom panel). The two-dimensional view of the communities depicts the relative size of the communities (number of residues) as colored circles of varying areas and the relative interconnectivity strength as lines of varying thicknesses. (**B**) Residues displaying the largest edge betweenness.

**Figure 10:**
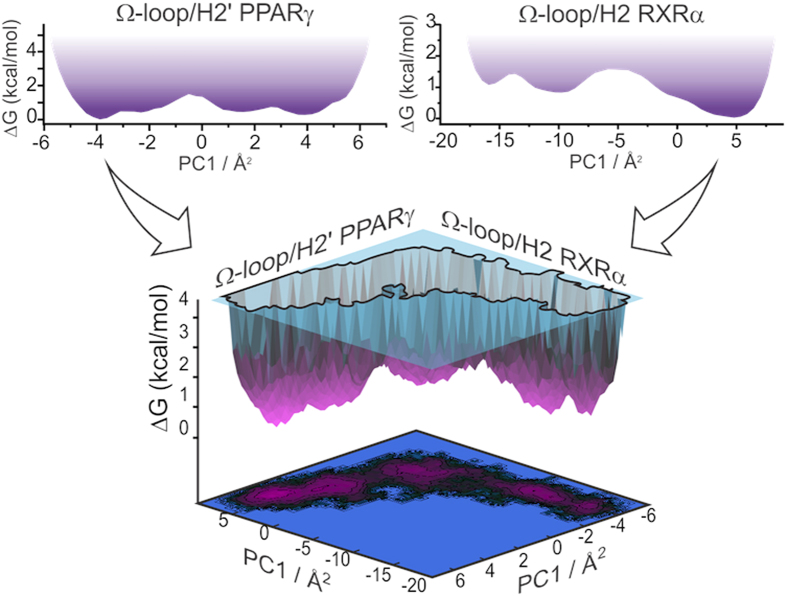
Two-dimensional energy landscapes corresponding to the dynamics of the two Ω-regions projected along their principal modes of motion (PC1). Flexible regions such as the Ω-loops correspond to flat areas in one-dimensional landscapes (top panels) that combine to form multidimensional canyons (bottom panels). Such canyon topography means that the conformation adopted by the RXRα Ω-loop restricts the range of conformations that can be adopted by the PPARγ Ω-loop and vice-versa.

The Girvan-Newman algorithm is an iterative procedure in which the edge with the highest betweenness is removed from the network and the betweenness of the remaining edges is recalculated, with communities being progressively isolated up to the point when each node will represent a community. The best division network can be determined using the modularity parameter^74^ in such way that nodes within a community are highly intra-connected while different communities are poorly inter-connected through few critical edges. The modularity values fall in the range from 0 to 1, with larger values indicating higher community structure quality. The optimum community structure obtained for the full-length complex (see Fig. 9) has modularity equal to 0.67, in agreement with standard modularity values found in 3D structure of proteins (0.4–0.7)^74^.

The choice of the *distance* and *frames cutoff* parameters is made using a criterion that guarantees convergence of the optimal community network, i.e. the Community Repartition Difference (CRD) defined as


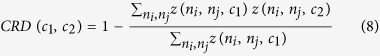


where *z* (n_i_, n_*j*_, c_*i*_) is 1 if nodes n_*i*_ and n_*j*_belong to the same community in a given partition c_*i*_ (or community structure) 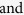 0 otherwise. The CRD provides a normalized count of pairs that are grouped together in two community structures, providing a good estimate of the similarities between different network partitions, as in the case of community structures obtained with different cutoff values.

## Additional Information

**How to cite this article**: Ricci, C. G. *et al*. Allosteric Pathways in the PPARγ-RXRα nuclear receptor complex. *Sci. Rep*. **6**, 19940; doi: 10.1038/srep19940 (2016).

## Supplementary Material

Supplementary Information

## Figures and Tables

**Table 1:** Residues with high correlation scores and demonstrated (or suggested) importance for PPARγ function.

Residue	Location	Evidence supporting functional importance
I267	Ω-loop	I267A abolishes 15d-PGJ_2_ activation (ref. 43)
R357	Loop6-7	Forms salt bridge that locks a ligand exit pathway (ref. 54)
P467	H12	P467L attenuates ligand binding and coactivator recruitment (ref. 55)
E471	H12	Participates in the charge clamp (ref. 29)
