# Supplementary material for: Allosteric Pathways in the PPARγ-RXRα nuclear receptor complex
Source: Sci Rep. 2016 Jan 29;6:19940. doi: 10.1038/srep19940 (PMC4731802; doi:10.1038/srep19940)
Supplement: Supplementary Information [file srep19940-s1.doc]

**Supplementary Information**

**Allosteric Pathways in the PPARγ-RXRα nuclear receptor complex**

*Clarisse G. Ricci^a^, Rodrigo L. Silveira^a^, Ivan Rivalta^b,c^, Victor S. Batista^c^, Munir S. Skaf ^a^*

^a^Institute of Chemistry, University of Campinas-UNICAMP, Cx. P. 6154, Campinas SP 13084-862, Brazil.

^b^Université de Lyon, CNRS, École Normale Supérieure de Lyon, UMR 5182, 46 Allée d’Italie, 69364 Lyon, Cedex 07, France.

^c^Department of Chemistry, Yale University, P.O. Box 208107, New Haven, CT 06520-8167, United States.

**Convergence Study and extended simulation time**

To address the important issue of results convergence within the 40 ns time window and to characterize the changes in correlated motions if longer MD simulations (around 100ns) would be used, we have: i) extended the simulation time of the three MD trajectories reported in the main text from 40ns to 120ns; and ii) considered three additional independent simulations of 100ns; for a total simulation time of 0.66 microseconds. Since the results and the conclusions reported in the main text are essentially based on the generalized correlation coefficients obtained by averaging three independent 40 ns MD simulations, we have calculated the average correlation matrices along the 0.66 microseconds trajectories and compared with the average matrix used in the main text results.

Figure S1 shows the correlation analysis based on the 0.66 microseconds MD simulations and the comparison with the average matrix used in the main text results (Fig. S1a). In particular, by selecting 27 windows of 40ns out of the entire set of trajectories (i.e. taking a 40ns window every 20 ns along the 0.66 microseconds trajectories) we have calculated the average correlation matrix associated with a 40 ns time window (Fig. S1b) and calculate the difference with respect originally reported correlation matrix obtained out of the 3 independent 40 ns MD simulations (Fig. S1a). Notably, this differential map (Fig. S1d) shows only small and homogeneous changes of the correlation coefficients, with an overall RMSD=0.024862. This indicates that the MD results based on the correlations coefficients reported in the main text are robust and converged for a time window of 40 ns.

To understand if the motions in the first 40 ns are those crucial for determining the allosteric mechanism, as assumed in the main text, we have analyzed the differences between the correlated motions in the 40ns and the 100ns time windows. We have, therefore, calculated the average correlation matrix associated with 6 windows of 100 ns along the entire 0.66 microseconds trajectories (Fig. S1c) and compared it with: i) the average matrix associated with the 27 windows of 40 ns; and ii) the original correlation matrix obtained out of the 3 independent 40 ns MD simulations. Both differential maps (Fig. S1e and S1f, respectively) indicate a homogeneous and minor decrease of correlations going from 40ns to 100ns, with RMSDs equal to 0.147168 and 0.160958, respectively. The homogeneity of the correlation shifts in these differential maps, i.e. the absence of regions with very large differences in correlated motions, indicate that the largest part of the correlations that govern the allosteric mechanism are already obtained with sampling in the 40 ns time window.


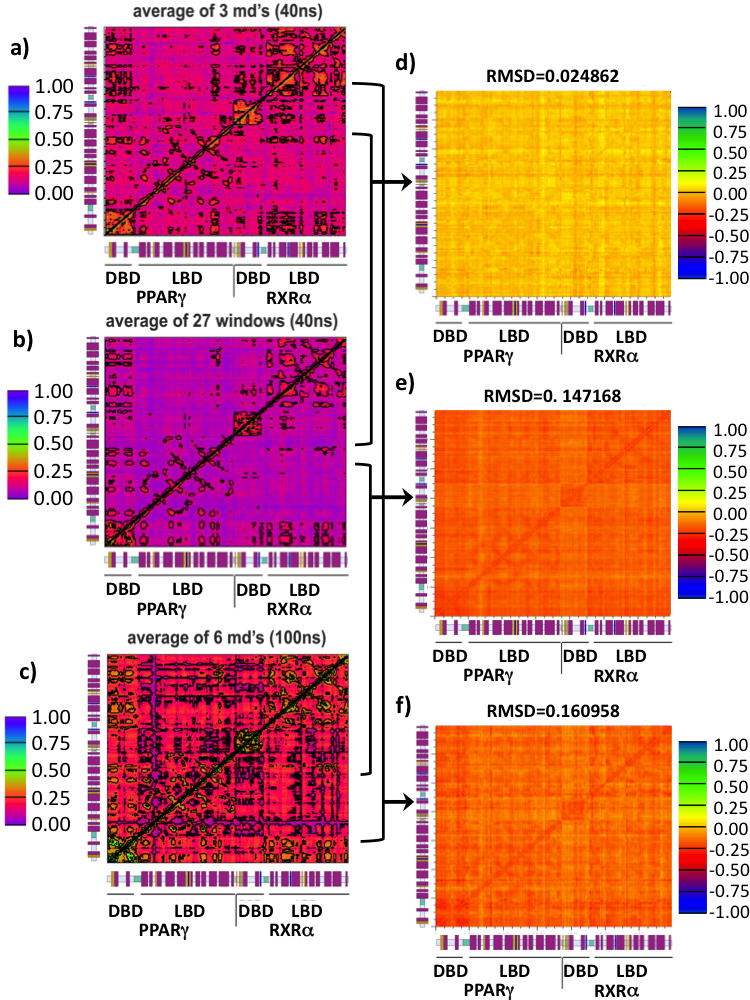


**Figure S1**. Correlation matrix of GC coefficients of the PPARg-RXRa complex, averaged over: (a) the three independent MD simulations of 40 ns, (b) the 27 windows of 40ns out of the entire set of trajectories (i.e. taking a 40ns window every 10 ns along the 0.66 microseconds trajectories), (c) the six independent simulationd of 100 ns; and the corresponding differential matrices, with panels e=a-b, e=b-c and f=a-c.


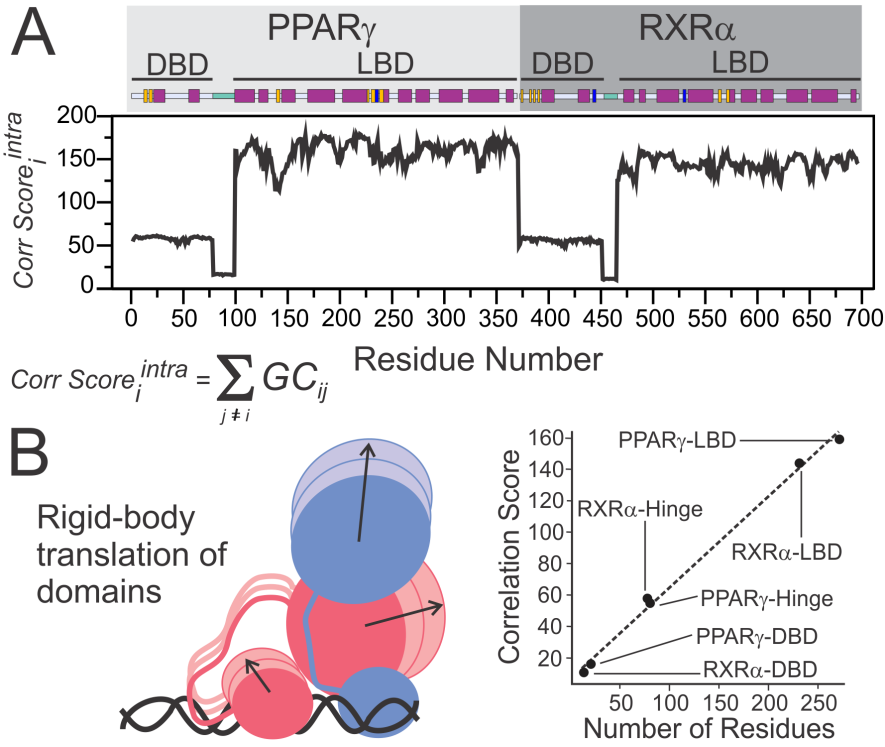


**Figure S2**. A) Distribution of intra-domain correlation scores. The observed plateaus are a result of domain translations, whereas fluctuations reflect deviations from a perfect rigid-body behavior. B) Scheme of domain translations taking place in the complex (left panel) and correlation between the size of domains (number of residues) and their accumulated intra-domain correlation scores (right panel).


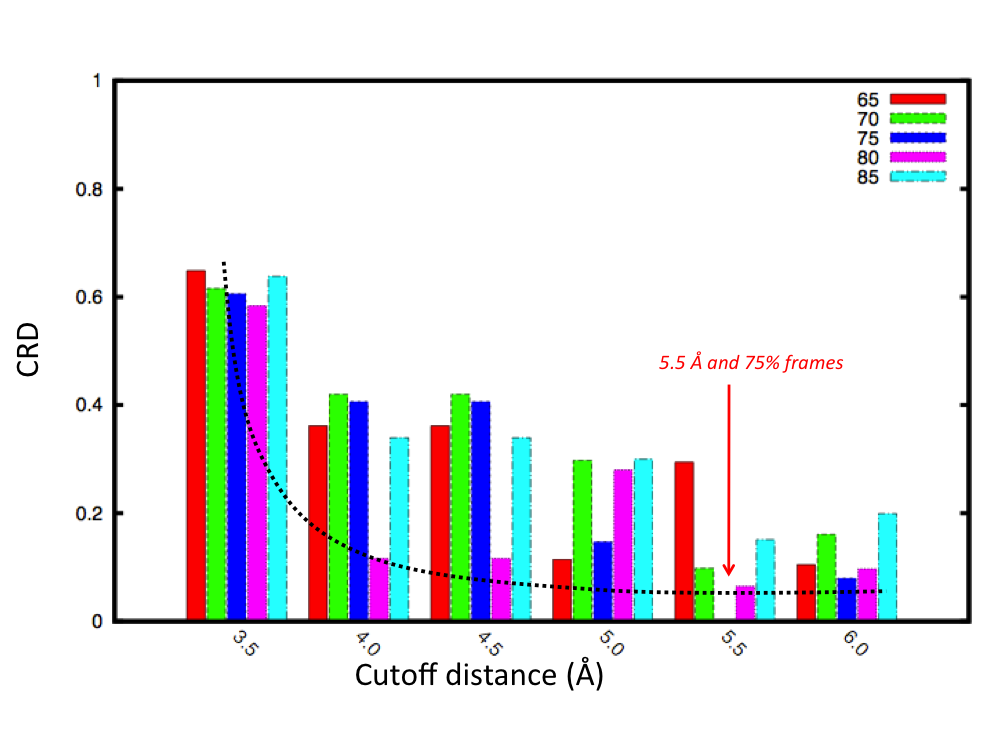


**Figure S3**. Community Repartition Differences (CRD) calculated for the full-length complex community structures obtained using a reference community structure with 5.5 Å and 75% cutoffs. The distance cutoff (from 3.5 to 5.5 Å) defines a contact in each frame of the simulation and the percentage cutoff (from 65% to 85%) is the percentage of frames the contact has to be formed. CRD equal to 0 if two community structures are identical and 1 if they are totally different.
